# Supplementary material for: Large-Scale Synthesis of Semiconducting Cu(In,Ga)Se2 Nanoparticles for Screen Printing Application
Source: Nanomaterials (Basel). 2021 Apr 28;11(5):1148. doi: 10.3390/nano11051148 (PMC8147049; doi:10.3390/nano11051148)
Supplement: Supplementary file 1 [file nanomaterials-11-01148-s001.zip › nanomaterials-1180917-supplementary.pdf]

nanomaterials

Supporting Information

Large-Scale Synthesis of Semiconducting $Cu(In,Ga)Se_2$ Nanoparticles for Screen Printing Application

Bruna F. Gonçalves [1,2,3], Alec P. LaGrow [1], Sergey Pyrlin [2], Bryan Owens-Baird [4,5], Gabriela Botelho [3], , Luis S.A. Marques [2], Marta M.D. Ramos [2], Kirill Kovnir [4,5], Senentxu Lanceros-Mendez [2,6,7] and Yury V. Kolen'ko [1,*]

1 International Iberian Nanotechnology Laboratory, 4715-330 Braga, Portugal; bruna.goncalves@inl.int (B.F.G.); alec.lagrow@inl.int (A.P.L.)
2 Center of Physics, University of Minho, 4710-057 Braga, Portugal; pyrlinsv@fisica.uminho.pt (S.P.); lsam@fisica.uminho.pt (L.S.A.M.); marta@fisica.uminho.pt (M.M.D.R.); senentxu.lanceros@bcmaterials.net (S.L.-M.)
3 Center of Chemistry, University of Minho, 4710-057 Braga, Portugal; gbotelho@quimica.uminho.pt
4 Department of Chemistry, Iowa State University, Ames, IA 50011, USA; bowens@iastate.edu (B.O.-B.); kovnir@iastate.edu (K.K.)
5 Ames Laboratory, U.S. Department of Energy, Ames, IA 50011, USA
6 BCMaterials, Basque Center for Materials, Applications and Nanostructures, UPV/EHU Science Park, 48940 Leioa, Spain
7 Ikerbasque, Basque Foundation for Science, 48009 Bilbao, Spain
* Correspondence: yury.kolenko@inl.int

Figures

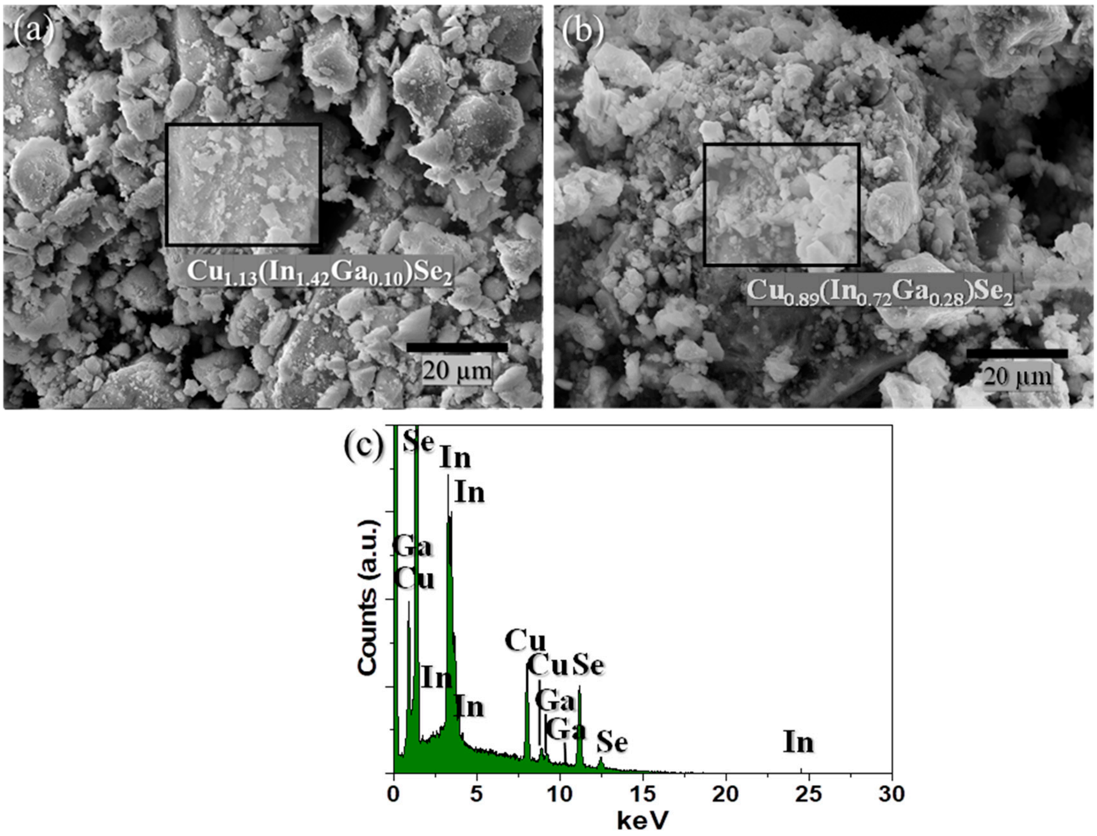

Figure S1. Scanning electron microscopy images of **Sample I** (**a**) and **Sample II** (**b**), and EDX spectrum of **Sample II** (**c**).

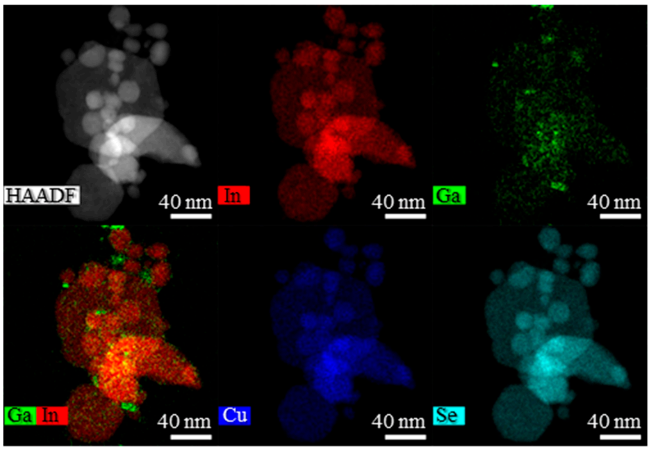

Figure S2. HAADF–STEM image and corresponding STEM–EDX elemental maps of **Sample I**.

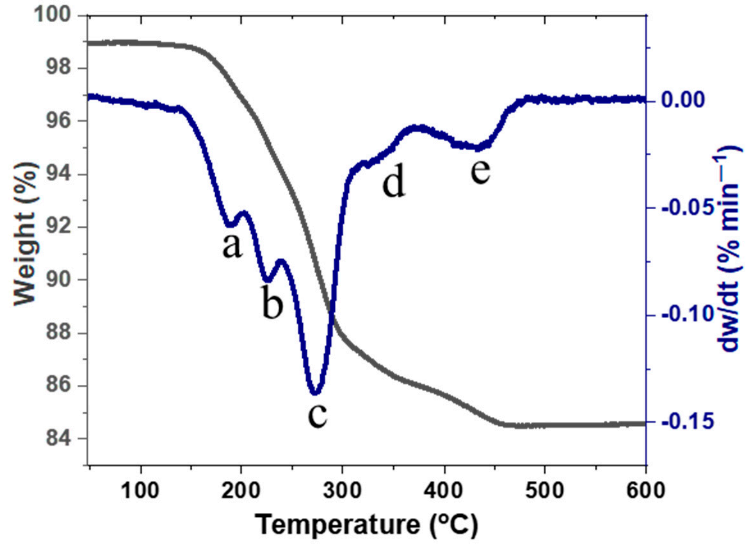

Figure S3. Thermogravimetric analysis with the corresponding derivative curve for the NPs from **Sample II** under Ar. Peak temperatures a: 187 °C, b: 226 °C, c: 273 °C, d: 337 °C, and e: 433 °C.

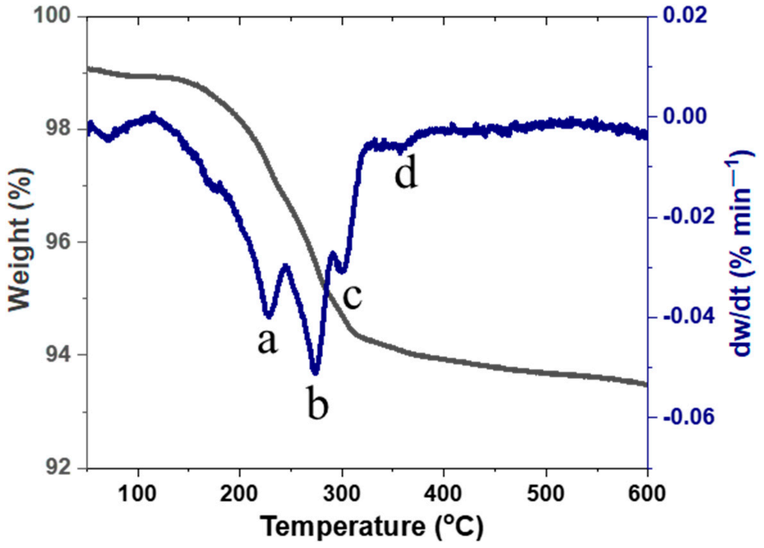

Figure S4. Thermogravimetric analysis with the corresponding derivative for the NPs from **Sample II** after ligand exchange under Ar. Peak temperatures a: 227 °C, b: 274 °C, c: 299 °C, and d: 357 °C.

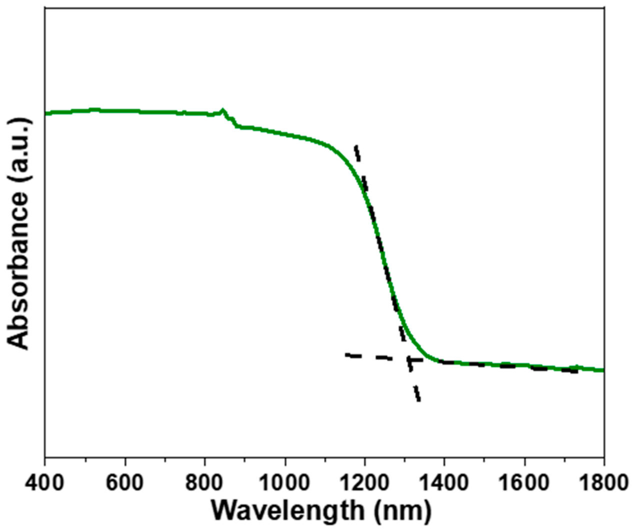

Figure S5. UV–Vis–NIR absorption spectrum of the synthesized NPs.

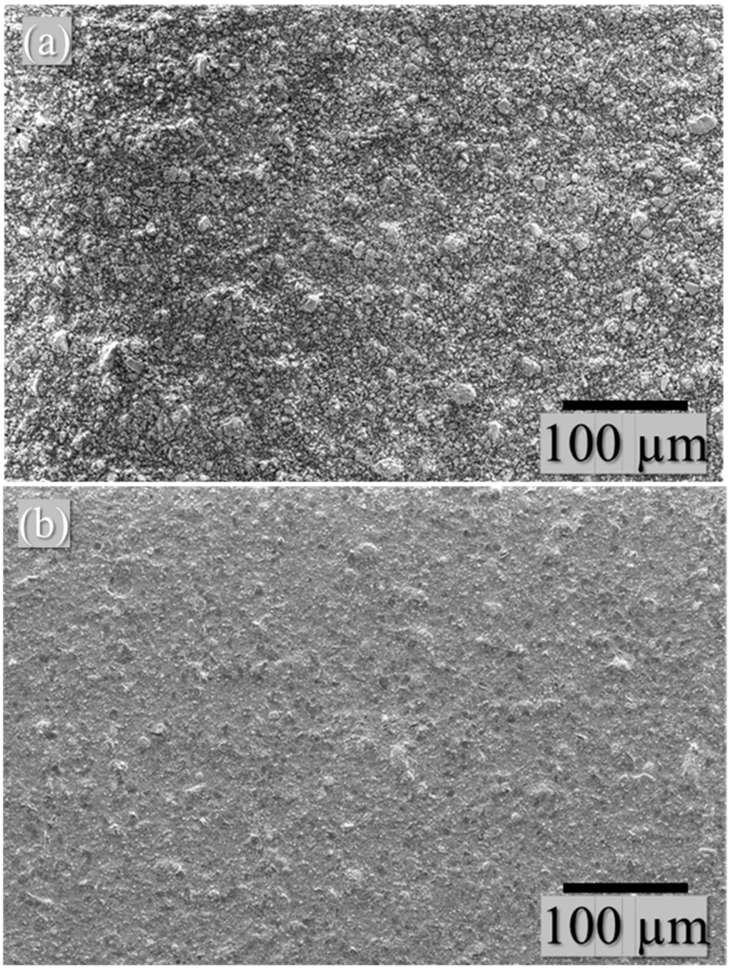

Figure S6. SEM surface images from screen-printed films of **Sample II** before (**a**) and after (**b**) wet ball milling.

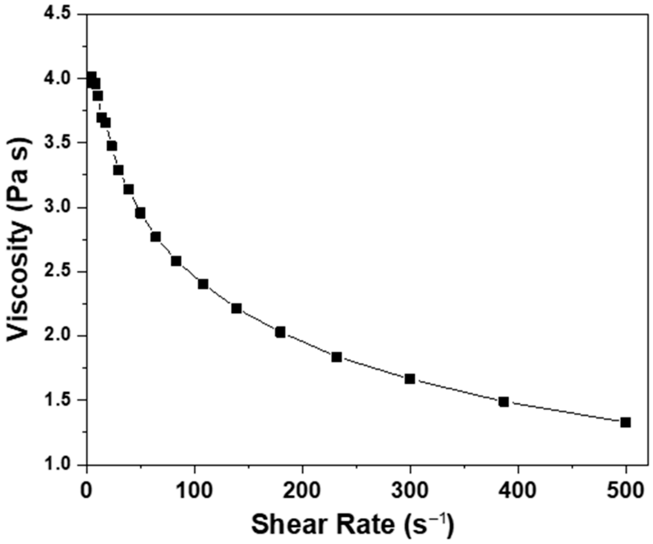

Figure S7. Dynamic viscosity of the formulated NP ink showing a non-Newtonian behavior.

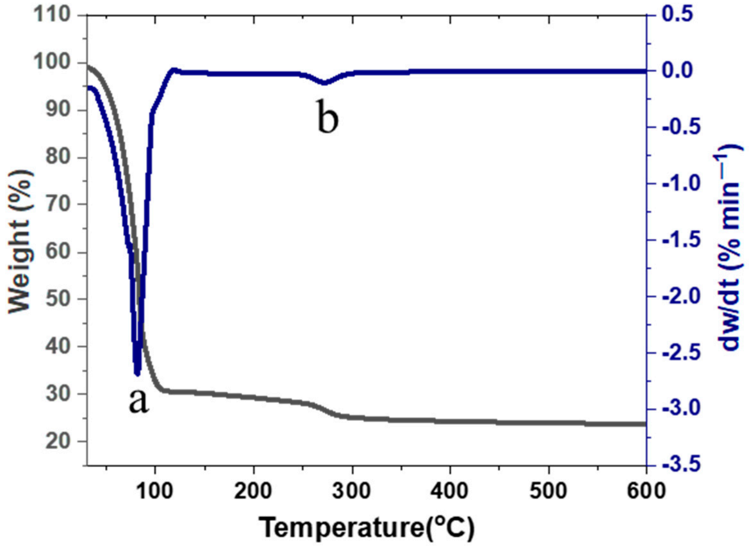

Figure S8. Thermogravimetric analysis with the corresponding derivative curve for the screen printable ink under Ar. Peak temperatures a: 81 °C and b: 271 °C.

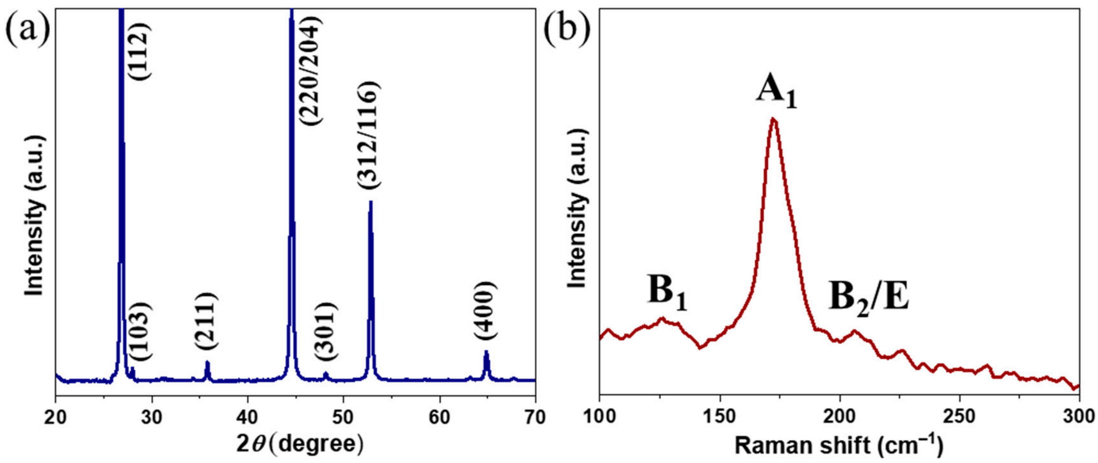

Figure S9. The experimental powder XRD pattern (**a**) and Raman spectrum (**b**) of the screen-printed film after annealing at 500 °C. In XRD figure, the *hkl* peak assignment is based on ICDD card no. 00-066-0140 for chalcopyrite.
